# ZNF139 increases multidrug resistance in gastric cancer cells by inhibiting *miR-185*

**DOI:** 10.1042/BSR20181023

**Published:** 2018-09-05

**Authors:** Bibo Tan, Yong Li, Qun Zhao, Liqiao Fan, Dong Wang

**Affiliations:** Department of General Surgery, The Fourth Hospital of Hebei Medical University, Shijiazhuang City, Hebei Province 050011, China

**Keywords:** drug resistance gene, gastric cancer, microRNA 185, multidrug resistance, zinc finger protein 139

## Abstract

It has been reported that the expression of zinc finger protein 139 (ZNF139) and *microRNA-185* (*miR-185*) were associated with proliferation, drug resistance of gastric cancer (GC) cells. However, the detailed mechanisms have not been fully investigated. The expression of ZNF139 in both GC tissues and cell lines was tested, then SGC7901/ADR or SGC7901 cells were transfected with ZNF139-siRNA, *miR-185* analog, or pcDNA-ZNF139. Cell activity was determined by MTT assay. Real-time PCR and Western blot were utilized to detect ZNF139, *miR-185*, and multidrug resistance (MDR) related genes including MDR1/P-gp, GST-π, MRP-1, Bcl-2, TS and Bax. ChIP and dual luciferase activity assay were used to investigate regulation between ZNF139 and *miR-185*. Increased ZNF139 and decreased *miR-185* expression were detected in GC tissues and cell lines. Transfection with ZNF139-siRNA into SGC7901/ADR cells markedly increased expression of *miR-185*, and treating with chemotherapeutic drugs ADR, 5-FU, L-OHP, the survival rate of SGC7901/ADR cells obviously decreased after ZNF139-siRNA transfection. On the other hand, transfection with pcDNA-ZNF139 in GC cell line SGC7901 resulted in an increased expression level of ZNF139 and a decline in the expression level of *miR-185*, meanwhile drug resistance of GC cells was clearly enhanced to ADR, 5-FU, L-OHP. Dual luciferase activity assay demonstrated that ZNF139 inhibited transcriptional activities of *miR-185*’s promoter in cells transfected with the reporter plasmid encompassing the upstream promoter region of *miR-185* along with pcDNA-ZNF139. Our data reveal that ZNF139 might promote MDR gene *MDR1/P-gp, MRP-1* and *Bcl-2* by prohibiting *miR-185*.

## Background

Gastric cancer (GC) is one of the most common malignancies in digestive tract worldwide with highest incidence and mortality rates [1,2]. Despite the advances in GC screening and detection methods in the past decades, the occurrence of GC remains high in China [3,4]. Currently, surgical removal of lesions is still the main treatment for GC; however, given that most patients are diagnosed at advanced stage, the outcome of surgical approach is limited [5,6]. Thus, chemotherapy plays a key role in eradicating malignant cells [7,8], however, the outcome is still not satisfactory with a 5-year survival rate less than 30% [9]. This is mainly attributed to multidrug resistance (MDR) in GC cells. MDR in GC is the main reason for chemotherapy failure, which leads to high mortality rate in GC patients [10–12]. So it is of great urgency to explore novel and potential therapeutic strategies in order to address MDR in GC patients. Many studies have been conducted and some of them have identified a few key molecules and signaling pathways that may be involved in GC MDR.

Zinc finger protein 139 (ZNF139), one member of the zinc finger protein family, can transcribe and regulate the downstream genes. van Dekken et al. [13] discovered that the expression of ZNF139 increased in cancer tissues of the gastro–esophageal junction and may be related to cancer growth and development, and the same research team also revealed that ZNF139 has participated in the process of gastric cells differentiation [14], promoting invasion and development of GC cells [15]. Furthermore, the increased expression of ZNF139 is also closely related to GC MDR [16]. However, the main downstream target of ZNF139 in GC MDR is still unknown. Therefore, to understand the mechanism of GC MDR, further investigation is required to determine the mechanism of ZNF139.

Over the past few years, the role of miRNAs has been a hotspot in cancer research. Studies have shown that *miR-185* is closely related to cancer growth, invasion and metastasis [17,18]. Also, there was a research exploring the role of *miR-185* in cisplatin-resistant human ovarian cancer cells [19]. All these evidences suggested that *miR-185* may be involved in GC MDR. However, the specific regulatory mechanism has not been fully understood. In the present study, we aimed to investigate the connection between ZNF139 and *miR-185*, and their functions and molecular mechanism in GC MDR.

## Materials and methods

### Patients and tissue specimens

A total of 35 patients with GC were enrolled at the General Surgery Department of The Fourth Hospital of Hebei Medical University. Participants included 23 males and 12 females, aged 42 –76 years (mean age: 61.8 ± 12.1 years) and their diagnosis was pathologically confirmed postoperatively. There were 21 well-differentiated and 14 poorly differentiated carcimoma in these participants. According to eighth edition UICC classification, there were three patients in stage I, seven in stage II, twenty in stage III, and five in stage IV. All participants were not treated with preoperative radiotherapy and chemotherapy. Each cancerous tissue and para-carcinoma tissue (approximately 1.0 cm × 0.5 cm × 0.5 cm) was obtained from each participant (over 3 cm from resection margin), and the specimens were immersed into liquid nitrogen for quick freezing and later transferred to −80°C cryogenic refrigerator for stock. Consent for the present study was obtained from all of the participants.

### Cell lines and main reagents

Human gastric cell line SGC7901 was obtained and cultured in Research Center, The Fourth Hospital of Hebei Medical University. GES-1 cell line was obtained from Shanghai Institutes of Biochemistry and Cell Biology, CAS. Adriamycin-resistant GC MDR cell line SGC7901/ADR was a gift from academician Fan Daiming in Digestive Disease Research Institute of The Fourth Military Medical University. RPMI 1640 culture medium and trypsin were purchased from Gibco Company; TRIzol reagent and Lipofectamine™ 2000 transfection reagent were obtained from Invitrogen. Fluorescence quantitative RT-PCR reagents and Reverse Transcription Kit were purchased from Promega. PCR primers and siRNA were synthesized at the Shanghai Sangon Biotech. Protein extraction kit was purchased from Beyotime Company, China; antibody for ZNF139 was provided by Sigma, U.S.A.; MDR1/P-gp, GST-π, MRP, Bcl-2, TS and β-actin antibodies were purchased from Santa Cruz Biotechnology, U.S.A.; MTT was bought from Sigma, U.S.A.

### Cell culture and transfection

Human GC cell lines SGC7901, SGC7901/ADR and GES-1 cell line for control were all cultured in RPMI 1640 (Invitrogen) containing 10% FBS, 100 mg/ml streptomycin and 100 U/ml penicillin. Specifically, SGC7901/ADR was cultured in 0.4 mg/l ADR for maintenance of the drug-resistance phenotype. This treatment was ceased 1 week prior to experiment. After incubation in a humidified 5% CO_2_ atmosphere at 37°C, cells were digested with 0.25% trypsin solution supplemented with 0.02% EDTA for subculturing. Design ZNF139-siRNA sequence by employing BLOCK-iT™ RNAi Designer (sequence: 5′-ACCTCGGAAGATTCAGCAT-3′), which would be transfected into GC cell lines SGC7901/ADR with high expression of ZNF139, with cells transfected with non-specific siRNA sequence (NS-siRNA: 5′-GACGAGTTGACTGCGATTG-3′) transfected serving as a negative control. Human ZNF139 overexpression vector were constructed. Coding sequence of human ZNF139 (NM_003439) was subcloned into pcDNA3.1 vector using KpnI and NotI restriction sites.

GC cells were seeded in six-well plates for 24 h prior to transfection, with a density of 4 × 10^5^/ml. The plasmid vector, siRNA or *miR-185* mimics were transfected into GC cells or resistant cells in accordance with the manual for the reagent transfection Lipofectamine™ 2000, of which cells were rinsed with RPMI 1640 to be serum-free and antibiotic-free. The transfection efficiency was detected 24 h later, and the subsequent experiments were also conducted.

### MTT assay

GC tissues as well as normal para-carcinoma tissues were cut into pieces and then ground. Subsequently, single cell suspension was prepared by means of filtering with 300 copper mesh. GC cells after digestion with 0.02% EDTA-0.25% trypsin were seeded into 96-well plates at a density of 5 × 10^4^ cells/ml, and ZNF139-siRNA was transfected as well as chemotherapeutic drugs (ADR, 5-FU, L-OHP) were added when cells grew to 80%. Each group set up six paralleled wells. Twenty microliters of MTT at a concentration of 5 mg/ml was added into the wells 4 h before the end of the experiment. The culture medium was discarded afterward. One hundred and fifty microliters of DMSO was added to each well, and absorbance value (OD value) was measured at a wavelength of 490 nm with a microplate reader after the plate being shaken for 15 min at room temperature. The above experiments were replicated for three times.

### RNA isolation and qRT-PCR

TRIzol methods were used to extract total RNA. Two microliters of RNA samples were incubated with RNase-free DNase at 37°C for 30 min, 65°C inactivation for 10 min and then were subjected to reverse transcription for template cDNA. Relative mRNA levels were measured using PCR. *GAPDH* served as a reference gene. A final volume of 20 μl PCR reaction was established according to instructions: 2 μl reverse transcription product, 10 μl SYBR Green Mix (Applied Biosystems, Foster City, CA), each 0.5 μl for the downstream primer (10 μmol/l). PCR parameters: 95°C for 5 min, and then three steps, 94°C, 30 s, denaturation; 60°C, 30 s, annealing; for 45 cycles. The primer sequences designed by Primer 5.0 and blasted for specificity are as follows: ZNF139: (F) 5′-CTTCCTGAGTTCTTGGTTTCG-3′ and (R) 5′-CCTTTGACCCACTGGTTTATG-3′; MDR1: (F) 5′-GAATGTTCAGTGGCTCCGAG-3′, (R) 5′-ACAATCTCTTCCTGTGACACC -3′; GST-ð: (F) 5′-ATACCATCCTGCGTCACCTG-3′, (R) 5′-TCCTTGCCCGCCTCATAGTT-3′; MRP1: (F) 5′-CATCAGCAGGCACCACAAC-3′, (R) 5′-TTCCAGGTCTCCTCCTTCTTG-3′; Bcl-2: (F) 5′-TGTGTGGAGAGCGTCAACC-3′, (R) 5′-TGGATCCAGGTGTGCAGGT-3′; TS: (F) 5′-TTTCTGACGGCAACTTCAAC-3′, (R) 5′-AGTCCAATGTCCAGCCCAT-3′; Bax, (F) 5′-TTTCTGACGGCAACTTCAAC-3′, (R) 5′-AGTCCAATGTCCAGCCCAT-3′; GAPDH: (F) 5′-GACCCCTTCATTGACCTCAAC-3′, (R) 5′-CGCTCCTGGAAGATGGTGAT-3′. The 2^−ΔΔ*C*^_t_ method was used to calculate quantitative PCR results. *GAPDH* was employed as the reference gene.

### Western blot analysis

Tissue and cell samples lysate was prepared using the lysis buffer: 1% Triton X-100, 150 mM NaCl, 10 mM Tris/HCl, pH 7.4, 1 mM EDTA, 1 mM EGTA, pH 8.0, 0.2 mM Na_3_VO_4_, 0.2 mM PMSF, and 0.5% NP-40. The same amount of protein samples separated by 10% polyacrylamide SDS gels (SDS/PAGE) were electrotransferred on to a PVDF membrane (Amersham Pharmacia Biotech). Membranes were blocked with 5% BSA for 2 h, followed by incubation with the primary antibody overnight at 4°C, and then with a horseradish peroxidase–conjugated secondary antibody for 2 h. Target bands were detected with an ECL detection kit (Santa Cruz, U.S.A.). *β-actin* acted as the internal control protein. The experiment was repeated three times.

### ChIP assay

ChIP assays were performed as following: in brief, cells were cultured in 1% formaldehyde at room temperature for 15 min for cross-linking of associated protein with DNA. Subsequently, the cross-linking was terminated due to the supplementation of glycine to a final concentration of 0.125 M. Cell lysis was initiated with 300 μl of radioimmune precipitation assay buffer (50 mM Tris/HCl, pH 8.0, 150 mM NaCl, 5 mM EDTA, 1% NP-40, 0.5% deoxycholate, and protease inhibitors). The resulting lysates were sonicated to generate chromatin fragments of approximately 600 bp, followed by assessment using agarose gel electrophoresis. The supernatants were centrifugated for 10 min at 13000 rpm, followed by incubation with 30 μl of protein A-Sepharose beads (Sigma, U.S.A) and sheared salmon sperm DNA for pre-clearance for 15 min at 4°C. After centrifugation at 13000 rpm for 5 min, the supernatants collected were divided into three shares equally: one for extraction of DNA as input, and the other for immunoprecipitation by rocking overnight at 4°C with or without 2 μg ZNF139 antibody. Then, protein A-Sepharose beads and sheared salmon sperm DNA were used to precipitate the immune complexes. After centrifugation, the beads were collected and rinsed twice sequentially with radioimmune precipitation assay wash buffer I (20 mM Tris/HCl, pH 8.1, 150 mM NaCl, 0.1% SDS, 1% Triton X-100, and 2 mM EDTA), wash buffer II (20 mM Tris/HCl, pH 8.1, 500 mM NaCl, 0.1% SDS, 1% Triton X-100, and 2 mM EDTA), wash buffer III (10 mM Tris/HCl, pH 8.1, 0.25 M LiCl, 1% NP-40, 1% deoxycholate, and 1 mM EDTA), and wash buffer IV (10 mM Tris/HCl, pH 8.1, and 1 mM EDTA). Two hundred microliters of elution buffer (1% SDS, 0.1 M NaHCO_3_) was added to the washed beads to elute the immunoprecipitants, which were incubated at 65°C overnight. The supernatants were centrifugated at 12000 rpm for 10 min to extract DNA by phenol–chloroform extraction and ethanol precipitation. PCR was conducted to amplify the promoter segments containing ZNF139-binding site.

### Luciferase assays

Cells grown to 70% confluence were transfected in triplicate with pGL3-*miR-185*-luc (1.5 kb upstream promoter region of *miR-185* were subcloned into pGL3-Basic vector), pcDNA-ZNF139 or pGL3-Basic, coupled with pRL-TK. After transfection for 48 h, the Dual-Luciferase® Reporter Assay System (Promega, Madison, WI) was used to measure the luciferase activity in accordance with the manufacturer’s manual. The relative luciferase activities in comparison with the luciferase activities of pRL-TK were presented as mean ± S.E.M.

### Statistical analysis

Results were all recorded, and data (expressions of ZNF139, *miR-185*, and chemosensitivity of cells) are presented as the mean ± S.D. ANOVA analysis and Dunnett’s test are processed utilizing SPSS 21.0 software. Then connection between ZNF139 and *miR-185*, and their relationship with drug resistance were all explored. *P*-value <0.05 was considered as statistical significance.

### Ethics approval and consent to participate

Ethics approval was given by Ethics Committee of The Fourth Hospital of Hebei Medical University for tissues application in the present study.

## Results

### ZNF139 and *miR-185* are expressed differentially in GC tissues

**Figure 1:**
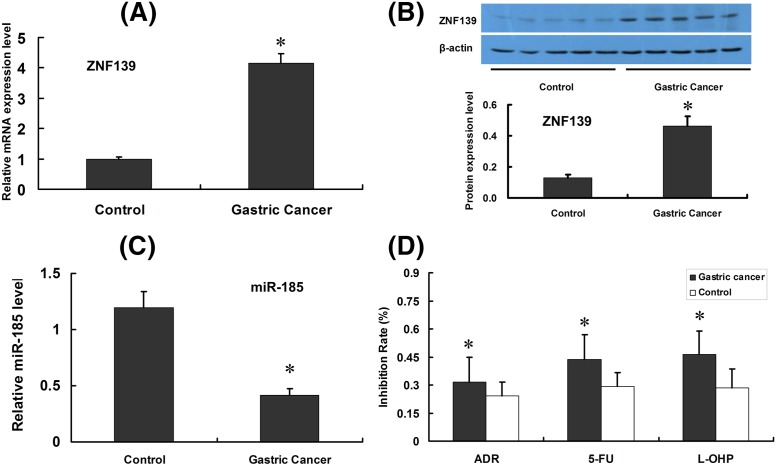
Expression of ZNF139, *miR-185* and chemosensitivity in GC tissues and para-cacinoma tissues Clinical samples of GCs and para-cacinoma tissues (Control group) were tested with qRT-PCR and Western blot, and expression of ZNF139 was shown as in (**A,B**). Expression of *miR-185* was tested with qRT-PCR (**C**). GCs and para-cacinoma tissues were also subjected to MTT assay to determine chemosensitivity to ADR, 5-FU, L-OHP (**D**). *P<0.05 compared with control group (para-carcinoma tissues).

Expression of ZNF139 was detected via qRT-PCR and Western blot in both GC and gastric para-carcinoma tissues, and the cellular sensitivity of L-OHP was determined by MTT. ZNF139 was up-regulated in GC tissues (Figure 1A,B, qRT-PCR as well as Western blot), compared with that in gastric para-carcinoma tissue (*P*<0.05). However, expression of miR-185 was reduced in GC tissue (*P*<0.05) (Figure 1C, qRT-PCR results). MTT assay revealed lower cell inhibition rate in GC than that in gastric para-carcinoma tissue when ADR, 5-FU and L-OHP were supplemented with the single-cell suspension of tissue (*P*<0.05) (Figure 1D).

### ZNF139 and *miR-185* are differentially expressed in gastric mucosa epithelial cell line and GC cell line

**Figure 2:**
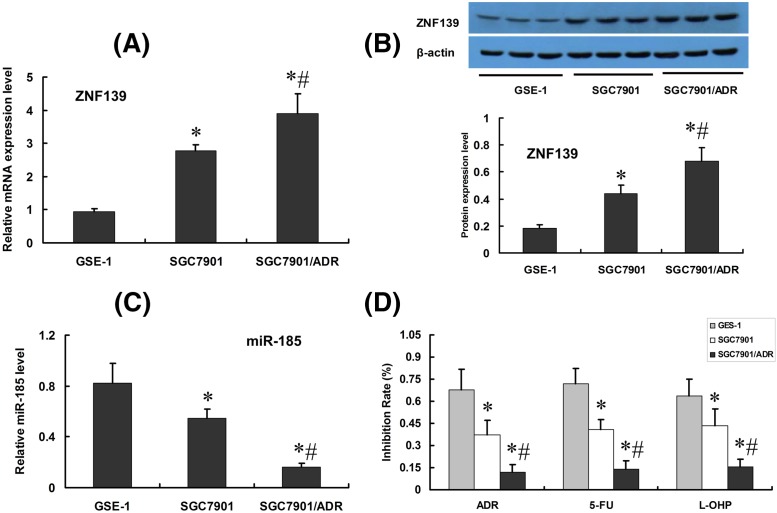
Expression of ZNF139, *miR-185* in different gastric cell lines GES-1, SGC7901 and SGC7901/ADR cell lines were subjected to qRT-PCR (**A**) and Western blot (**B**) assays to determine the expression of ZNF139. The relative expression levels of *miR-185* were shown in (**C**). Cell lines were also subjected to MTT assay to determine chemosensitivity to ADR, 5-FU, L-OHP (**D**) *P<0.01 compared with GES-1 group; ^#^*P*<0.01 compared with SGC7901 group.

Amongst three cell lines (SGC7901, SGC7901/ADR and GES-1), SGC7901/ADR indicated the highest ZNF139 expression, followed by SGC7901, and the lowest in gastric mucosa epithelial cell line (*P*<0.05) (Figure 2A,B, qRT-PCR as well as Western blot). Meanwhile, the expression of *miR-185* showed the opposite tendency: SGC7901/ADR the least, SGC7901 followed, and GSE-1 the highest (*P*<0.05) (Figure 2C, qRT-PCR results). MTT assay indicated that with the addition of ADR, 5-FU and L-OHP, SGC7901/ADR acquired the lowest cell survival rate, SGC7901 group followed, and GES-1 group was the highest (*P*<0.05) (Figure 2D, histogram results).

### ZNF139 regulated drug resistance of SGC7901/ADR cells

**Figure 3:**
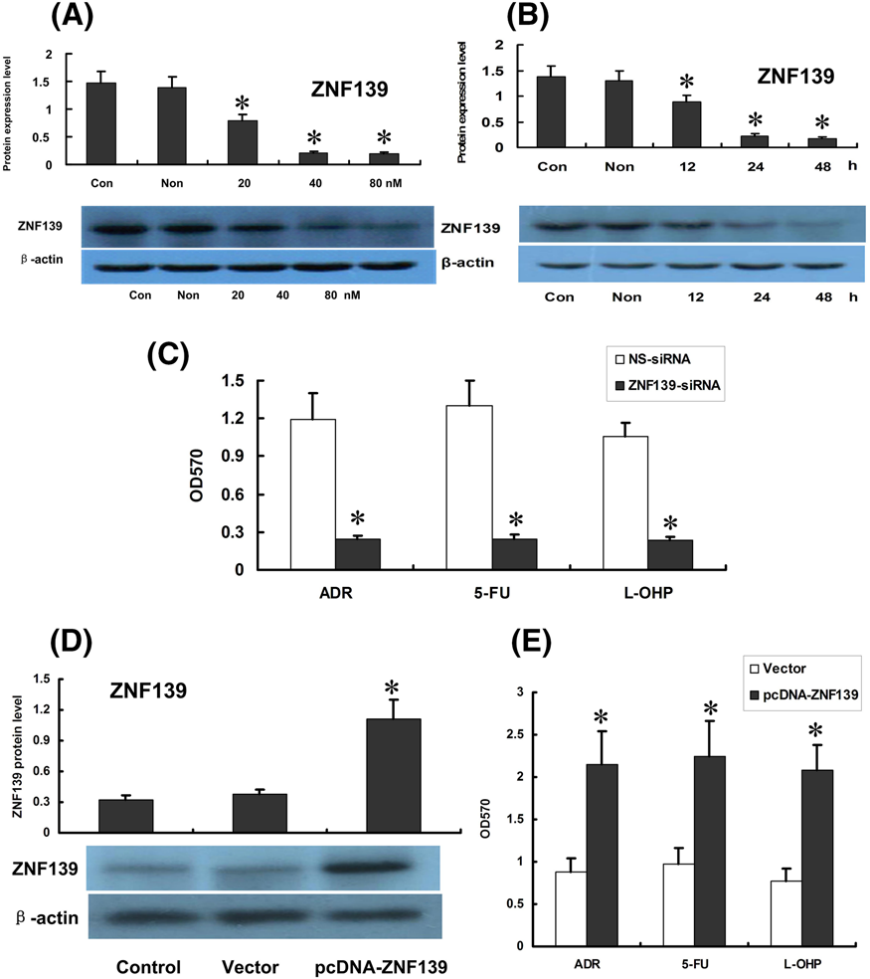
Effect of ZNF139-siRNA on ZNF139, *miR-185* expression and chemosensitivity of SGC7901/ADR cells Expression of ZNF139 were identified by Western blot as well as qRT-PCR ((**A**) for different doses and (**B**) for different times). Then chemosensitivity to ADR, 5-FU, L-OHP were tested in ZNF139-siRNA group, NS-siRNA group (**C**). The expression of ZNF139 was remarkably increased in SGC7901 after transfecting with pcDNA-ZNF139 48 h later (*P*<0.05) (**D**). Chemosensitivity to ADR, 5-FU, L-OHP in Vector group and pcDNA group was shown as in (**E**). *P<0.01 compared with NS-siRNA group, Vector group, control group.

Western blot demonstrated that ZNF139 remained the same level in cells after it was transfected with control-siRNA while mRNA and protein of ZNF139 reduced with varying degrees in SGC7901/ADR cells transfected with ZNF139-siRNA. In that process, ZNF139 appeared to decline the most with a percentage of 90% (*P*<0.05) (Figure 3A). ZNF139 decreased in a dose-dependent manner. In addition, 48 h after transfection showed a maximum inhibitory effect (*P*<0.05) (Figure 3B). Then, MTT assay illustrated that cell survival rates were significantly reduced with ADR, 5-FU and L-OHP in cells SGC7901/ADR transfected with ZNF139-siRNA compared with non-transfected cells (*P*<0.05) (Figure 3C). Also it was found that expression of ZNF139 was remarkably increased in SGC7901 after transfecting with pcDNA-ZNF139 48 h later (*P*<0.05) (Figure 3D). After transfection with pcDNA-ZNF139, activity of cells treated with ADR, 5-FU or L-OHP increased significantly (*P*<0.05) (Figure 3E).

### Effects of *miR-185* on MDR of drug-resistant SGC7901/ADR cells

**Figure 4:**
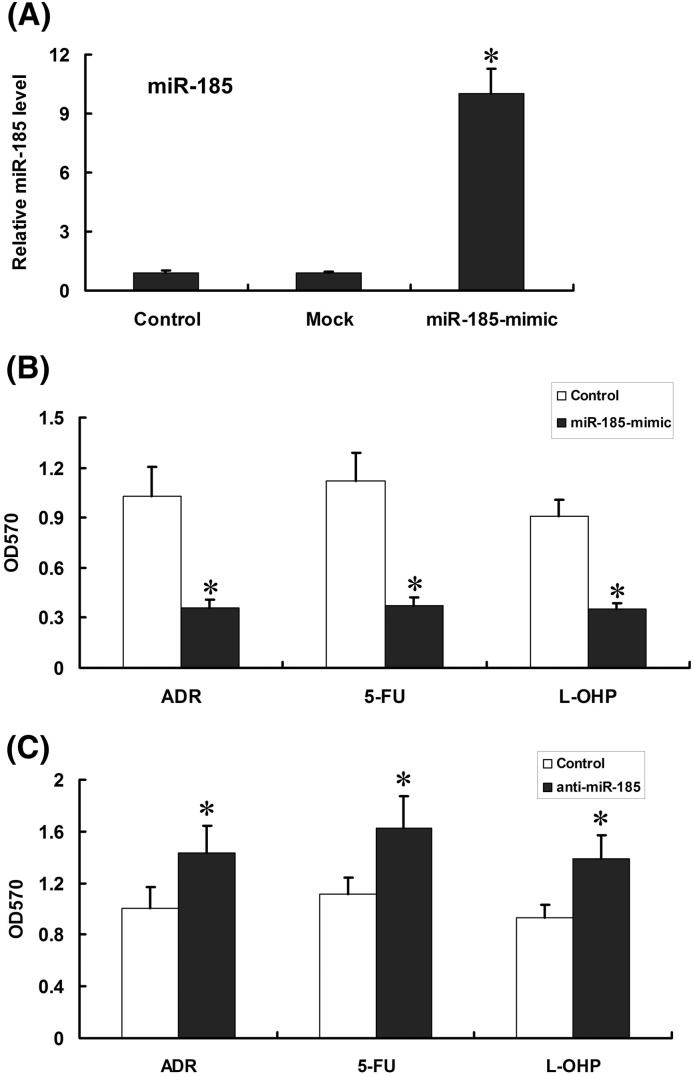
Effect of *miR-185* mimics on *miR-185*, ZNF139 expression and chemosensitivity of SGC7901/ADR cells Cells were transfected with *miR-185* mimics or Mock, and the expression of *miR-185* was tested with qRT-PCR (**A**) and chemosensitivity to ADR, 5-FU, L-OHP were tested in *miR-185* mimics group, control group (**B**). Anti-*miR-185* was transfected into SGC7901/ADR cells, and variation of chemosensitivity was detected with MTT (**C**). *P<0.01 compared with Mock group, control group.

Expression of *miR-185* was up-regulated in SGC7901/ADR when *miR-185* mimics was transfected into the drug-resistant GC cells (*P*<0.05) (Figure 4A). Based on MTT assay, cell survival rate of SGC7901/ADR was obviously reduced after transfection of *miR-185* mimics (*P*<0.05) (Figure 4B). Anti-*miR-185* was also employed to inhibit *miR-185* in SGC7901/ADR cells, then cell survival rate increased significantly after transfection (Figure 4C).

### ZNF139 inhibits transcription of *miR-185* in SGC7901 cells

**Figure 5:**
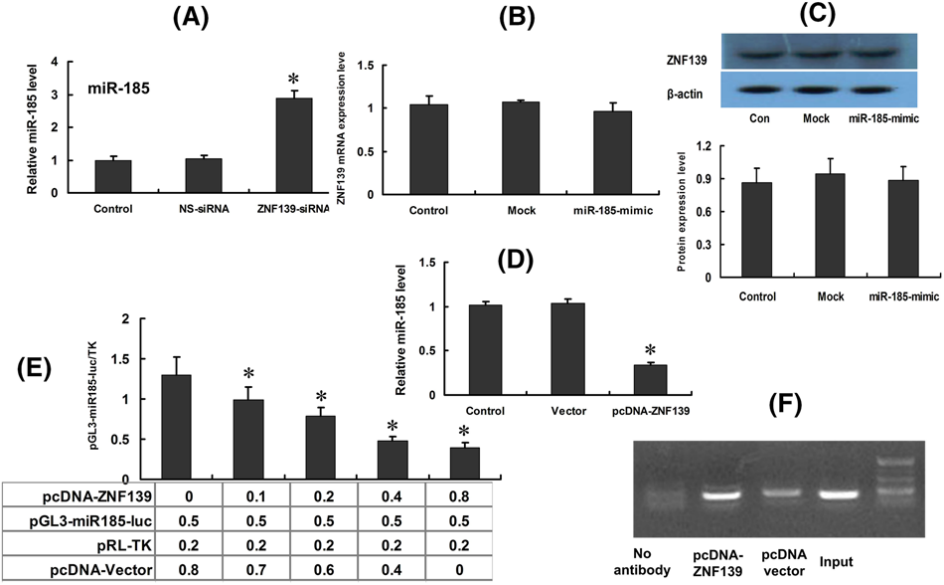
Effect of ZNF139 on the transcription of *miR-185* in SGC7901 cells Expression of *miR-185* were tested with qRT-PCR after ZNF139 was inhibited in SGC7901/ADR cells (**A**). Meanwhile, there was no remarkable change in the expression of ZNF139 in SGC7901/ADR after transfection ((**B**,**C**) qRT-PCR and Western blot). After transfection with pcDNA-ZNF139, the expression of *miR-185* was detected (**D**). The expression levels of *miR-185* were affected by the concentration of ZNF139 transfection through the luciferase assays (**E**). ChIP showed the physical interaction between ZNF139 and *miR-185* (**F**). *P<0.01 compared with Vector group, control group.

The expression of *miR-185* was significantly increased after ZNF139-siRNA was transfected into cells SGC7901/ADR (*P*<0.05) (Figure 5A). Meanwhile, there was no remarkable change in the expression of ZNF139 in SGC7901/ADR after transfection (Figure 5B,C, qRT-PCR and Western blot). *miR-185* in SGC7901 cells was down-regulated significantly after transfecting with pcDNA-ZNF139 48 h later (*P*<0.05) (Figure 5D). ZNF139 was speculated to be a critical factor affecting *miR-185* transcription. We established a luciferase reporter gene plasmid of *miR-185* with the 2-kb sequence on the upstream of the promoter region, which would be co-transfected into cells along with pcDNA-ZNF139. Reporter gene analysis results showed that ZNF139 may prohibit the promoter activity of *miR-185* (Figure 5E). It was further confirmed that ZNF139 directly bound to the promoter region of *miR-185* by using ChIP analysis (Figure 5F). Thus, ZNF139, as a transcription factor, was shown to prohibit the transcription activity of mi-R185 in SGC7901 through direct coupling to the promoter region of *miR-185*.

### Inhibition of ZNF139 using siRNA suppresses expression of drug-resistance genes

**Figure 6:**
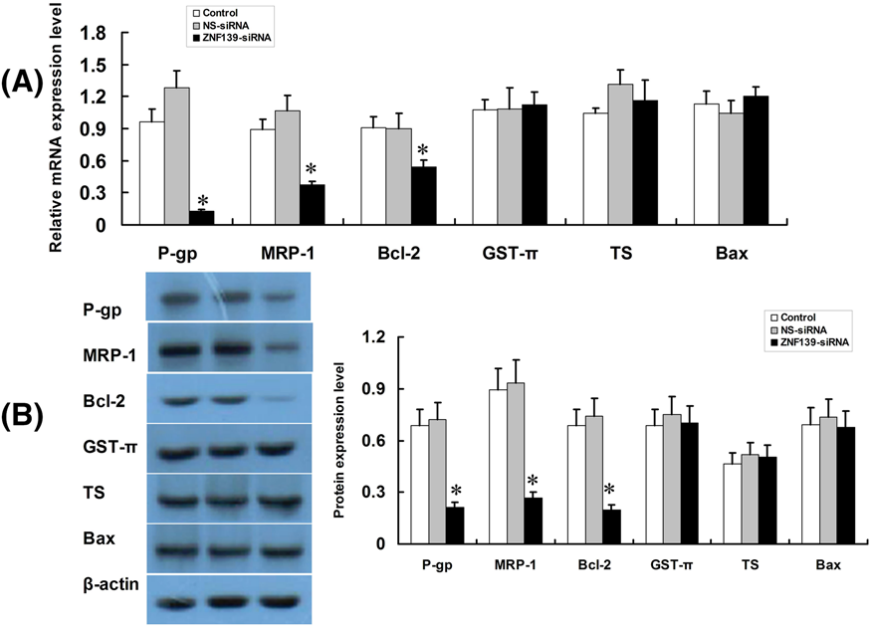
Effect of ZNF139 inhibition to P-gp, MRP1, Bcl-2, GST-π, TS, Bax in SGC7901/ADR cells After transfection with ZNF139-siRNA or NS-siRNA, SGC7901/ADR cells were subjected to qRT-PCR (**A**) and Western blot (**B**) assays to determine the expression of P-gp, MRP1, Bcl-2, GST-π, TS, Bax. Values were shown as mean ± S.D., for cell samples *n*=3 in each group. *P<0.01 compared with NS-siRNA group or control group.

The results showed that the expression of MDR1/P-gp, MRP-1, Bcl-2 in SGC7901/ADR were significantly suppressed due to the inhibition of ZNF139, whereas there was no meaningful change in the expression of GST-π, TS and Bax. Figure 6A was result of qRT-PCR and Figure 6B was result of Western blot (*P*<0.05).

### Inhibition of *miR-185* causes a reduction in drug resistance genes expression in SGC7901/ADR cells

**Figure 7:**
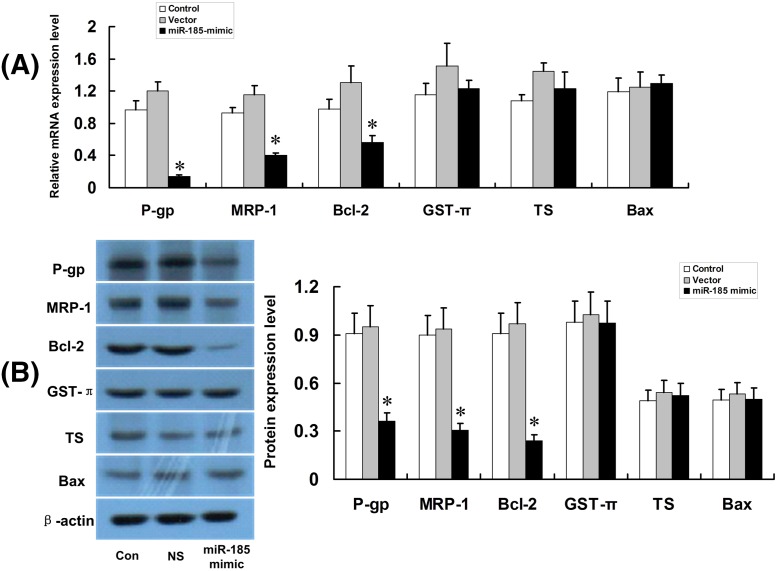
Effect of *miR-185* mimics on P-gp, MRP1, Bcl-2, GST-π, TS, Bax in SGC7901/ADR cells After transfection with *miR-185* mimics or Vector, SGC7901/ADR cells were subjected to qRT-PCR (**A**) and Western blot (**B**) assays to determine the expression of P-gp, MRP1, Bcl-2, GST-π, TS, Bax. Values were shown as mean ± S.D., for cell samples *n*=3 in each group. *P<0.01 compared with NS-siRNA group or control group.

The expression of MDR1/P-gp, LRP and Bcl-2 remarkably reduced after *miR-185* mimics sequence was transfected into GC7901/ADR cells (*P*<0.05), whereas changes were not noticed in the expression of GST-π, TS and Bax (*P*>0.05). Figure 7A was result of qRT-PCR and Figure 7B was result of Western blot.

### Up-regulation of ZNF139 with pcDNA-ZNF139 promotes expression of some drug-resistant genes

**Figure 8:**
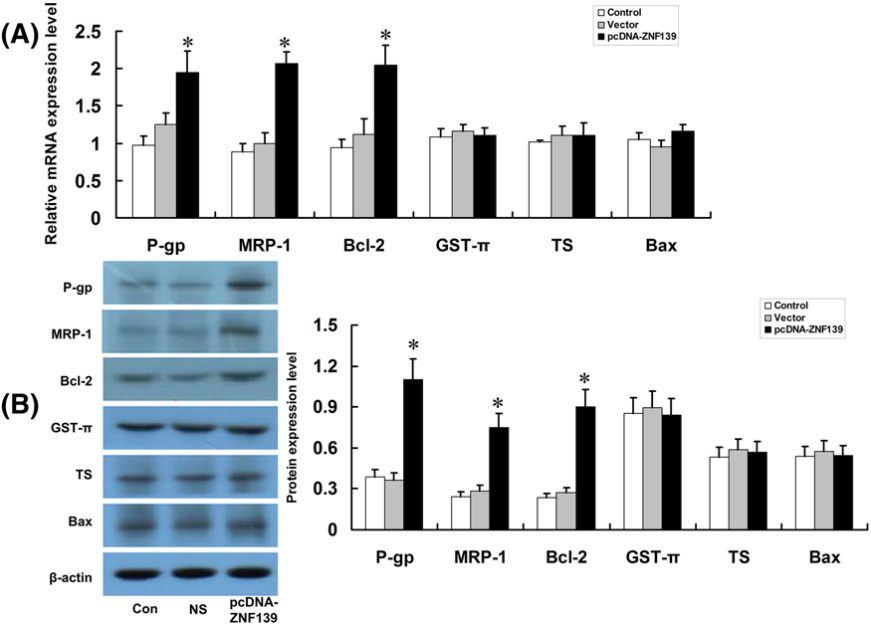
Effect of ZNF139 overexpressionon P-gp, MRP1, Bcl-2, GST-π, TS, Bax in SGC7901 cells After transfection with pcDNA-ZNF139 or Vector, SGC7901 cells were subjected to qRT-PCR (**A**) and Western blot (**B**) assays to determine the expression of P-gp, MRP1, Bcl-2, GST-π, TS, Bax. Values were shown as mean ± S.D., for cell samples *n*=3 in each group. *P<0.01 compared with NS-siRNA group, control group.

Results showed that the up-regulation of ZNF139 increased the expression of P-gp, MRP-1, Bcl-2 in SGC7901 cells and no significant changes were observed regarding the expressions of GST-π, TS and Bax (*P*<0.05). Figure 8A was result of qRT-PCR and Figure 8B was result of Western blot.

## Discussion

The incidence rate of GC ranks first amongst digestive malignances in China [20]. Moreover, the prognosis of GC is very poor [21]. MDR of GC cell directly contributes to the failure of chemotherapy. Previous data from our research indicated that ZNF139 participates in regulating MDR and can reverse the drug resistance of GC cell lines MKN28 [16]. Aiming to further explore the mechanism of ZNF139 on MDR in GC, in the present study we selected GC cell line SGC7901/ADR, which can stabilize characteristics of MDR in GC cell. Compared with normal gastric tissues and cell lines, GC tissues and cell lines showed higher expression of ZNF139. In addition, the MDR was stronger in GC tissues and its cell lines than in para-cancer tissues; expression of ZNF139 even higher in MDR cell line. Therefore, we hypothesize that ZNF139 promotes MDR in GC cells.

ZNF139 consists of six C2H2 zinc finger structures including SCAN and a KRAB domain. Zinc finger structure belongs to DNA-binding domain, while SCAN and KRAB promote the interaction of proteins between cofactors [22]. There have been a few reports about the relationship between ZNF139 expression and tumors. van Dekken et al. [13] discovered that the expression of ZNF139 was increased in adenocarcinoma of esophago–gastric junction. Also, ZNF139 can promote the invasion and progression of GC cells [15]. After using RNAi technique to suppress the expression of ZNF139, we found that gastric cell line MKN28 became more sensitive to chemotherapeutic drugs, and related drug-resistance genes varied [16]. All these studies demonstrated that ZNF139 is closely related to GC. But the mechanism of ZNF139 on GC MDR has not been fully understood. Therefore, we further explored the effect of ZNF139 on MDR of GC cells through gene interference and cloning techniques. Our data indicated that the inhibition of ZNF139 dramatically repressed drug resistance in gastric MDR cell lines. Then drug resistance were demonstrated markedly increased when the synthesized gene sequence for overexpression of ZNF139 was transfected into non-resistant GC cell lines. The findings indicated ZNF139 plays an important role in regulating MDR in GC.

MiRNAs refer to a class of small and widespread non-coding ssRNAs that consist of approximately 22 nts. Recent studies showed that MDR was closely associated with miRNAs in tumors [23–25]. *MiR-185* was recently discovered to be closely related to cancers. One research showed that low expression of *miR-185* has become an indicator of recurrence of hepatocellular carcinoma and poor prognosis [26]. Another research indicated that *miR-185* can suppress the growth of breast cancer, ovarian cancer, and hepatocellular carcinoma by inhibiting Six1 [27]. Our research also showed that transfection of *miR-185* analog increased MDR in GC cell SGC7901/ADR. The effects of the *miR-185* on MDR is still unclear, but our previous studies have shown some evidence and that effects will be discussed in our further study. Moreover, Li et al. [28] figured out that human ARC (apoptosis repressor with caspase recruitment domain) mRNA 3′-UTRs contained two binding sites for *miR-185*, and at both binding sites, *miR-185* was involved in regulating ARC expression. High ARC expression contributed to chemotherapy resistance in cancer cells by targetting the mitochondrial fission machinery. Those results provided evidence for our research. Further experiments in our study indicated that *miR-185* was negatively regulated by ZNF139 in GC tissue and cell lines: inhibition of ZNF139 increased *miR-185*. In contrast, overexpression of ZNF139 reduces expression of *miR-185*. These findings indicated regulation of ZNF139 in expression of *miR-185*. Further study demonstrated that *miR-185* mimics rescued drug-resistance characteristics in ZNF139 knockdown cells. Notably, ZNF139 directly binds and promotes *miR-185* transcription, revealing that ZNF139 regulates MDR of GC partially through *miR-185*.

The genes associated with MDR include MDR1/P-gp [29,30], MRP-1 [31,32], GST-π [33,34], Bcl-2 [35], TS [36,37] and Bax [38], before and after the intervention of ZNF139 in GC cells to delineate the regulating mechanism of ZNF139-*miR-185* pathway in GC MDR. In addition, it was demonstrated that the inhibition of ZNF139 attenuated expression of MDR1/P-gp, MRP and Bcl-2, while levels of their expression were increased dramatically after transfection of *miR-185* mimic. But the expression of GST-π, TS and Bax showed no obvious change before and after ZNF139, miR-185 interference. Accordingly, overexpression of ZNF139 markedly up-regulated the expression of MDR1/P-gp, LRP and Bcl-2 in GC cell line SGC7901. Hence, our data demonstrated that ZNF139 -miR-185 pathway causes MDR properties in GC by means of inducing MDR1/P-gp, LRP and Bcl-2.

## Conclusion

In all, our research demonstrated the abnormal expression of ZNF139 and *miR-185* in GC tissues and cell lines. ZNF139 was found to be an upstream regulator of *miR-185*. ZNF139 -*miR-185* pathway increases characteristics of MDR by inducing expression of MDR1/P-gp, MRP and Bcl-2 in GC, although the mechanism needs to be investigated further. In conclusion, these results suggest that ZNF139-*miR-185* pathway is crucial in initiating MDR in human GC and this pathway may also be employed as a novel therapeutic strategy in the treatment of GC MDR.

## Abbreviations

ADR: adriamycin
ARC: apoptosis repressor with caspase recruitment domain
5-FU: 5-fluorouracil
GC: gastric cancer
LRP: lung resistance protein
MDR: multidrug resistance
OD: optical density
P-gp: P-glycoprotein
RT-PCR: reverse transcription-polymerase chain reaction
ZNF139: zinc finger protein 139
